# Supplementary material for: Conditional chemoconnectomics (cCCTomics) as a strategy for efficient and conditional targeting of chemical transmission
Source: eLife. 2024 Apr 30;12:RP91927. doi: 10.7554/eLife.91927 (PMC11060718; doi:10.7554/eLife.91927)
Supplement: Figure 3—source data 3. [file elife-91927-fig3-data3.zip › Figure 3 source data3 part 2.zip!/Figure 3-Source data 3-part2/1052-13-CRISPResso_Report_ejedac2.zip!/CRISPResso_on_1052_16/2b.Nucleotide_percentage_quilt_around_sgRNA_agtggacaaactggtacatg.pdf]

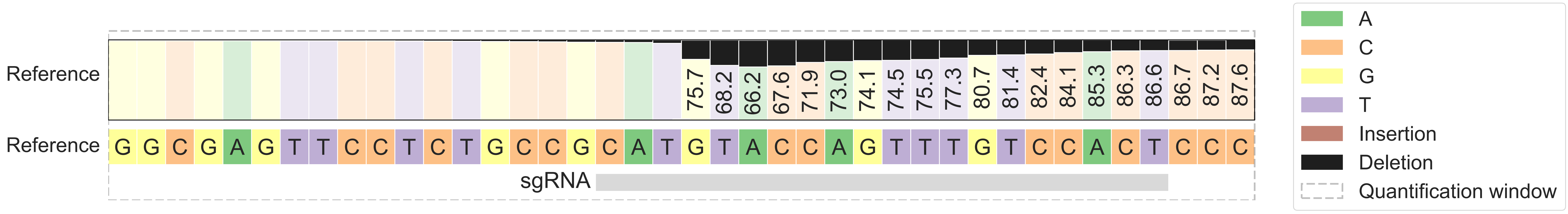

Reference
Reference
G G C G A G T T C C T C T G C C G C A T G T A C C A G T T T G T C C A C T C C C
75.7
68.2
66.2
67.6
71.9
73.0
74.1
74.5
75.5
77.3
80.7
81.4
82.4
84.1
85.3
86.3
86.6
86.7
87.2
87.6
sgRNA
A
C
G
T
Insertion
Deletion
Quantification window
